# Interspecific Habitat Suitability of Four Southeast Asian Spiny Climbing Palms (Korthalsia) Through Species Distribution Modeling

**DOI:** 10.3390/plants15091348

**Published:** 2026-04-28

**Authors:** Tushar Andriyas, Nisa Leksungnoen, Suwimon Uthairatsamee, Chatchai Ngernsaengsaruay, Nisachol Pungtambol, Pichet Chanton, Nittaya Mianmit, Wirongrong Duangjai, Buapan Puangsin, Phruet Racharak

**Affiliations:** 1 Department of Environmental Science, Faculty of Science, Chulalongkorn University, Bangkok 10330, Thailand; thugnomics28@gmail.com; 2 Department of Forest Biology, Faculty of Forestry, Kasetsart University, Bangkok 10900, Thailand; fforsmu@ku.ac.th (S.U.); nisachol.pu@ku.th (N.P.); fforprr@ku.ac.th (P.R.); 3 Department of Botany, Faculty of Science, Kasetsart University, Bangkok 10900, Thailand; fsciccn@ku.ac.th (C.N.); pichet.cha@ku.th (P.C.); 4 Department of Forest Management, Faculty of Forestry, Kasetsart University, Bangkok 10900, Thailand; ffornym@ku.ac.th; 5 Department of Silviculture, Faculty of Forestry, Kasetsart University, Bangkok 10900, Thailand; fforwrd@ku.ac.th; 6 Department of Forest Product, Faculty of Forestry, Kasetsart University, Bangkok 10900, Thailand; fforbpp@ku.ac.th

**Keywords:** species distribution model, statistical analysis, rattan, conservation status, non-timber forest products, climbing palms

## Abstract

Rattans of the genus *Korthalsia* are ecologically and economically important non-timber forest resources in Southeast Asia, yet their conservation is limited by knowledge of species-specific distribution patterns and environmental constraints. We modeled the potential distributions of four *Korthalsia* species (*K. flagellaris*, *K. laciniosa*, *K. rigida*, and *K. scortechinii*) using species distribution models (SDMs). Models were fitted in R using the sdm package, and ensemble maps were generated by combining predictions from Random Forest (RF), Generalized Linear Models (GLMs), Generalized Additive Models (GAM), and GLMnet. The top predictors influencing habitat distribution included soil physical structure, atmospheric moisture demand, and canopy light availability. The dominance of these factors reflects three distinct and non-interchangeable environmental axes that regulate belowground moisture dynamics, atmospheric constraints on gas exchange, and the energetic requirements for recruitment. All four species ensemble models significantly outperformed the null model, and spatial block cross-validation (k = 5, 200 km blocks) indicated a marginal drop in area under the curve (AUC) values, confirming a predictive signal under geographically independent evaluation. Ensemble suitability maps identified Peninsular Malaysia, Borneo, and Sumatra as centers of predicted habitat. Core habitat was estimated to be less than 0.6% of total suitable area for all species, ranging from 980 km^2^ (*K. scortechinii*) to 19,256 km^2^ (*K. rigida*), with anthropogenic modification exceeding 50% in the core habitat in *K. flagellaris* and *K. rigida*. These results provide the first species-specific baseline for these *Korthalsia* across Southeast Asia, supporting more targeted conservation and restoration planning under varying habitat constraints.

## 1. Introduction

The Southeast Asia region is a global biodiversity hotspot, with high levels of endemism and plant megadiversity [1,2]. The plant diversity in this region can be attributed to environmental factors such as high light intensity, stable year-round temperatures, and heavy rainfall, more so under the effects of climate change [3]. This diverse flora includes spiny climbing palms or rattans, which belong to the Calamoideae subfamily (Palmae family) and utilize cirri or flagella for climbing [4]. Calamoideae comprises approximately 550 species [5] that exhibit a climbing behavior, either solitary or clustered, with spiny stems and fruits covered in overlapping reflexed scales.

Within the Calamoideae subfamily is a genus of clustering climbing palms, known as *Korthalsia* Blume [5,6,7]. Recent surveys recognize the presence of 28 species of *Korthalsia* [5] in various parts of Southeast Asia, using records such as the Global Biodiversity Information Facility or GBIF database [8,9], indicating that this genus is endemic to Southeast Asia [10]. Many *Korthalsia* species have been reported to form mutualistic relationships with ants, which use the plant’s tubular ocrea (a sheath-like leaf structure at the stem nodes) as a domatium, providing a protected nesting space [11]. The *Korthalsia* species are frequently found in lowland and hill tropical rainforests and characterized by slender to moderately robust, spiny, high-climbing, and aerially branching hapaxanthic stems; catkin-like inflorescences; and monoecious reproductive systems. They have distinct pinnate leaves that have rhomboid (diamond-shaped) leaflets and a cirrus, while leaf sheaths lack knees [12].

According to the International Union for Conservation of Nature (IUCN), the conservation status of all *Korthalsia* species were either locally threatened [13] or not readily available. Chan et al. [14] reported that in Singapore, *K. flagellaris* Miq (or ant rattan) and *K. rigida* Blume were critically endangered, while *K. laciniosa* (Griff.) Mart. and *K. scortechinii* Becc. were presumed nationally extinct. Steady deforestation to increase land needed for agriculture can be attributed to the reduction of their natural habitat [15].

Climbing rattans, including the *Korthalsia* genus, are a valuable source of non-timber forest products, with traditional uses in basketry and binding, supporting the livelihoods of local communities dependent on forest resources [16,17,18,19]. The most widely distributed species is *K. laciniosa*, which is frequently found in Myanmar, the Andaman and Nicobar Islands, Thailand, Cambodia, Laos, Vietnam, Malaysia, Sumatra, Java, and the Philippines and is an integral part of the livelihood for ethnic tribes in the Andaman and Nicobar Islands [20]. The species has high market demand but is exported in limited quantity (3%) due to demand generated by local consumption [21]. *K. laciniosa* (Griff.) Mart. is used as a source of durable and flexible cane [22], with most rattan products still being sourced from wild-harvested plants, making their wild populations at increased risk of extinction. Lange [23] indicated that the lipophilic extracts of *K. rigida* stems primarily contained free acids, glycerides, and sterol esters.

Although considerable progress has been made in documenting the species richness and taxonomy of this genus, most of the existing research related to broad-scale geographic comparisons [24,25,26,27]. Species composition, distribution, and site overlap remain insufficiently explored, with previous studies contributing to checklists of *Korthalsia* in Peninsular Malaysia, but these revisions extend to include Sabah and Sarawak [28,29,30]. *K. flagellaris* is a distinct species given the bluish-green lanceolate leaflet with praemorse margin. It is the only species found in peat swamp forest at elevations between 3 and 50 m [12]. Recently, Ngernsangsaruay et al. [31] updated a comparative account of the vegetative morphology, distribution, ecology, conservation status, and traditional uses of the four *Korthalsia* species; however, the location was limited to only in Thailand.

This study examined four *Korthalsia* species that included *K. flagellaris*, *K. laciniosa*, *K. rigida*, and *K. scortechinii* to determine the most probable areas of their distribution based on significant environmental and soil factors in Southeast Asia, aiming towards their biodiversity conservation and management using species distribution modeling (SDM). The primary objective of this study were to: (i) calibrate and compare the discriminatory power of ecological models using an ensemble model for each species, identifying the most influential environmental and edaphic drivers shaping their distributions; (ii) test the statistical significance of fitted models by comparing performance against a null model; (iii) quantify potential suitable habitat extent by suitability class to provide spatially explicit estimates directly applicable to conservation planning; and (iv) assess anthropogenic pressure within predicted suitable habitat to identify species-specific priority areas for intervention within the context of local and regional biodiversity. The findings of this analysis refine the understanding of the distribution of these four *Korthalsia* species, inform the selection of probable planting areas, and support conservation status assessments in Southeast Asia. Additionally, this research complements studies on population diversity and developmental processes in the genus.

## 2. Results

### 2.1. Species Distribution Modeling

The environmental and soil conditions in Southeast Asia were determined using the terraclimate and ISRIC databases. After excluding collinear variables using the vif (variance inflation factor) function, using a threshold of five, the most important climate and soil variables (16 in number) reduced to nine, which included VPD; light intensity; evapotranspiration; bulk density; sand, silt, and clay percentages; nitrogen content; and elevation. These variables were used to generate the most probable spatial distribution for the species using SDM. Replication was incorporated using subsampling and bootstrapping with 30% test samples per model and three replicates per model. The training (Table S1) and testing performance of various SDMs, as determined through the fit metrics (AUC, COR, TSS, and deviance), indicated that GLM, GAM, GLMnet, and RF methods (listed in Table 1) were the best classifiers across the four species amongst the available methods in the SDM package and were used in the ensemble prediction of species distribution. RF had the highest AUC, COR, and TSS and the lowest deviance, indicating the highest classification accuracy among the various methods. To check if the high observed RF performance was inflated by spatial autocorrelation, a spatial block cross-validation (k = 5, block size = 200 km) was conducted (see Table S2). The spatial CV AUC values remained above 0.70 for the four species, with the drop in performance ranging from 0.17 (*K. scortechinii*) to 0.21 (*K. flagellaris*), which is consistent with the expected penalty for removing spatial autocorrelation between training and test partitions. The results indicated that RF predictions captured genuine species–environment relationships rather than overfitting to spatially clustered training data. Furthermore, null model significance testing confirmed that all four species distribution models performed significantly better than random expectation (Table S3, Figure S1). Full-model AUC values ranged from 0.84 (*K. rigida*) to 0.905 (*K. scortechinii*), substantially exceeding their respective null model means, validating that the ensemble predictions reflected genuine environmental signal rather than sampling artefacts or spatial autocorrelation alone.

**Figure 1:**
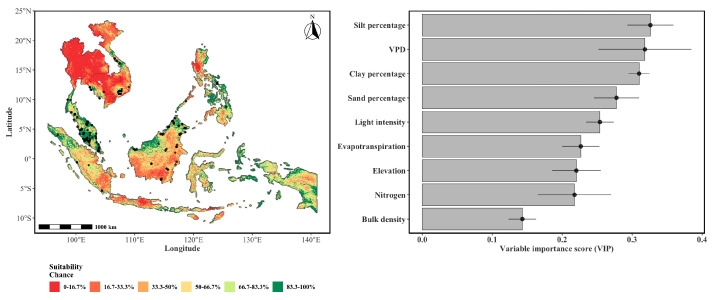
A composite habitat suitability for the genus *Korthalsia* in Southeast Asia. The map represents the cell-wise mean of the ensemble habitat suitability probability (range: 0–1) for four species: *K. flagellaris*, *K. laciniosa*, *K. rigida*, and *K. scortechinii*. Warmer colors indicate areas of higher predicted suitability chance for multiple species, identifying potential conservation hotspots for rattan diversity. The map was generated by creating an ensemble of the most influential species distribution models listed in Table 1. To enhance visual contrast and highlight spatial heterogeneity, suitability chance probabilities were binned into six percentile-based classes (0–16.7%, 16.7–33.3%, 33.3–50%, 50–66.7%, 66.7–83.3%, 83.3–100%), represented by a diverging color palette ranging from dark red to dark blue. The right panel lists the variable importance scores over various models.

Figure 1 contains the predicted spatial distribution of the four *Korthalsia* species using an ensemble model of GLM, GAM, GLMnet, and RF algorithms, which indicates distinct ecological patterns, with high probability (indicated by greener colors) concentrated primarily in parts of Peninsular Malaysia, Borneo, and Sumatra. It is to be noted that the predicted suitability areas across the *Korthalsia* species were characterized by moderate probability values. The absence of very high suitability values reflects the inherently conservative nature of ensemble predictions, where aggregation across multiple algorithms and replicated partitions reduces extreme outputs that are not consistently supported across all model runs. The probability gradient reflects a marked reduction in habitat suitability moving away from equatorial zones. The relative contribution of predictors was assessed using the permutation-based importance method within the SDM framework. For the ensemble model, variable importance scores (VIPs) were calculated as the mean decrease in accuracy when each variable’s values are randomly permuted.

Across the four species, silt percentage (0.326), VPD (0.318), clay percentage (0.310), sand percentage (0.277), and light intensity (0.254) were the top five most influential variables, collectively indicating that soil texture, atmospheric water demand, and canopy energy interception jointly constrained the overall species distributions. High VIP scores for all three soil textural classes suggests that edaphic properties exert a strong influence on habitat suitability relative to climatic variables, highlighting the role of soil physical characteristics in rattan ecology. The prominence of VPD reflected a sensitivity of these large-leaved climbing palms to atmospheric moisture deficit, mediating hydraulic stress and stomatal regulation under varying canopy conditions. Light intensity underscores the species’ dependence on canopy gaps for establishment and climbing, consistent with their ecology as shade-intolerant understory plants requiring periodic light penetration for recruitment. Notably, evapotranspiration was relatively less important than VPD, suggesting that the species response was influenced to a greater degree by instantaneous atmospheric demand than by integrated landscape water balance.

### 2.2. Interspecific Differences in Distribution

**Figure 2:**
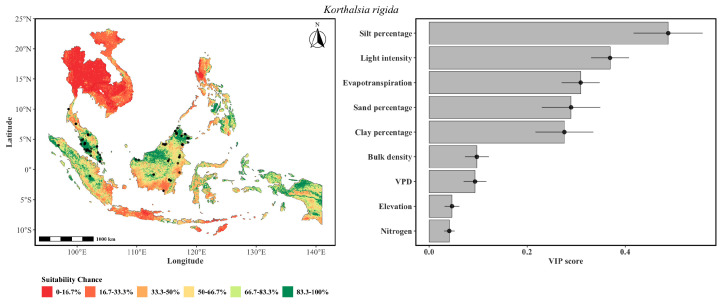
The probability of the spatial distribution of *K. rigida* as predicted by a weighted ensemble of GLM, GAM, GLMnet, and RF methods. The format of the figure is the same as that of Figure 1.

The distribution pattern of *K. rigida* (Figure 2) exhibited a pronounced ecological affinity toward the tropical lowlands of Peninsular Malaysia and Borneo, with a comparatively restricted predicted range relative to the genus-level composite. A high influence of silt indicated a strong preference for fine-textured soils that retain moisture while remaining sufficiently aerated. Light intensity ranked second, consistent with the species’ dependence on canopy gaps for establishment. The importance of evapotranspiration and sand percentage reflected sensitivity to landscape-scale water balance and soil drainage. However, VPD and bulk density exerted a lesser influence on the species distribution. A very low influence of elevation and nitrogen content suggests that *K. rigida* had a semblance of regional plasticity. Notably, light availability influenced model replicates, reinforcing the species obligate dependence on canopy disturbance for recruitment. This constraint indicated light as the nonnegotiable primary filter, followed by context-dependent edaphic and hydrologic factors.

**Figure 3:**
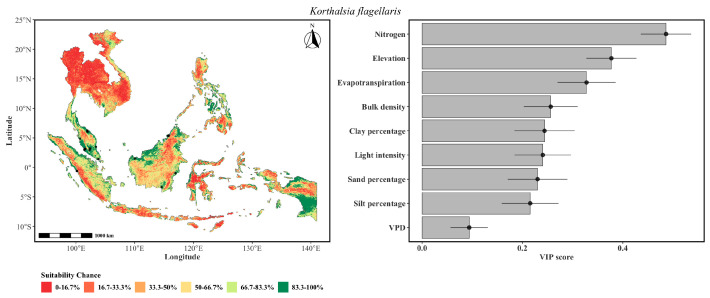
The probability of the spatial distribution of *K. flagellaris* as predicted by a weighted ensemble of GLM, GAM, GLMnet, and RF methods. The format of the figure is the same as that of Figure 2.

The predicted distribution of *K. flagellaris* (Figure 3) indicated an ecological preference for the lowland forests of Peninsular Malaysia, Sumatra, and parts of Borneo, with a notably fragmented pattern suggesting sensitivity to localized environmental filters. Nitrogen was the most influential variable, suggesting the presence of *K. flagellaris* being restricted to areas with elevated soil fertility, likely reflecting the high metabolic demands of a large-statured climbing palm that is invested heavily in leaf production and stem elongation. A strong influence of evapotranspiration suggests that water balance was a limiting factor primarily at the drier extremes of the species’ range. Light intensity and clay percentage had a comparable influence, indicating that canopy gaps and soil texture co-limit its establishment, while VPD exerted the lowest influence, distinguishing this species from its congeners that appear more sensitive to atmospheric moisture deficit. Notably, soil texture variables (sand, silt, clay) had lower influence, suggesting that edaphic constraints were geographically specific rather than universal.

**Figure 4:**
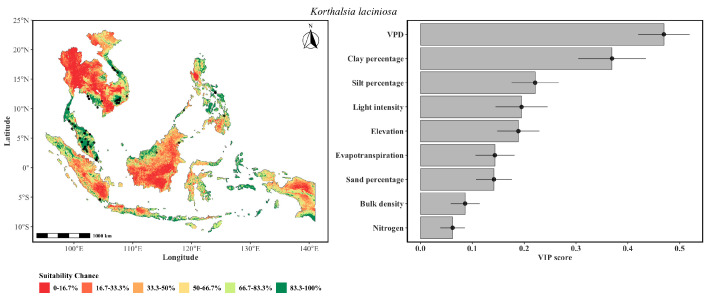
The probability of the spatial distribution of *K. laciniosa* as predicted by a weighted ensemble of GLM, GAM, GLMnet, and RF methods. The format of the figure is the same as that of Figure 2.

The predicted distribution of *K. laciniosa* (Figure 4) spanned Peninsular Malaysia, Northern Sumatra, and parts of Indochina, representing a broad range of occurrence. VPD was a dominant predictor, indicating that atmospheric moisture demand is a physiological filter for this species. The importance of clay percentage indicated that fine-textured soils with high water-holding capacity are essential for buffering the species against episodic moisture stress due to high evaporative demand. Light intensity and silt percentage had comparable influence, reinforcing the criteria of canopy light availability for climbing establishment alongside soil physical properties governing root zone aeration. Similar to *K. rigida*, an extremely low VIP for nitrogen distinguishes this species from its congeners with a broader range unconstrained by fertility.

**Figure 5:**
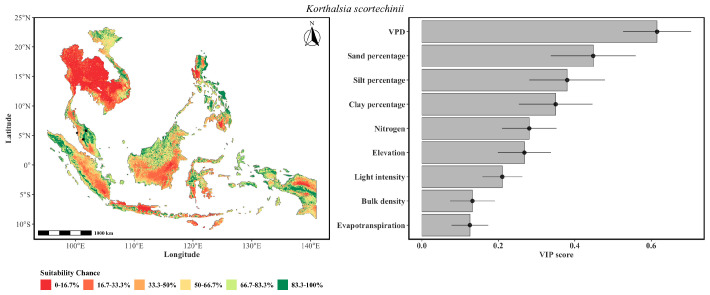
The probability of the spatial distribution of *K. scortechinii* as predicted by a weighted ensemble of GLM, GAM, GLMnet, and RF methods. The format of the figure is the same as that of Figure 2.

Given its sparse occurrence data, the predicted distribution of *K. scortechinii* (Figure 5) was restricted primarily to Peninsular Malaysia and central Sumatra, representing the most geographically constrained range among the four species. VPD was the dominant variable, indicating that this species occupies an exceptionally narrow atmospheric moisture regime. This extreme sensitivity to air dryness indicated that *K. scortechinii* was the most physiologically vulnerable of the four congeners. The influence of sand and silt percentage revealed a strong edaphic control, with the species requiring well-drained coarse-textured soils for root aeration while simultaneously depending on fine fractions for moisture retention, a seemingly contradictory requirement that likely restricts it to specific alluvial or sandstone-derived soil types where both conditions are present. The influence of clay percentage and nitrogen reinforced that soil fertility and water-holding capacity operate alongside texture as secondary filters. Notably, a very low influence of light intensity and evapotranspiration suggested that given the stringent VPD and soil texture requirements, canopy gaps and landscape water balance became less limiting.

The ensemble modeling predicted substantial marginal habitat across Southeast Asia for all four *Korthalsia* species (Table S4), ranging from approximately 3.14 to 3.18 million km^2^, with core habitat being a very small fraction of the total suitable area, consistently less than 0.6% for all species. The core habitats predicted for *K. flagellaris* and *K. scortechinii* were low at around 5563 km^2^ and 980 km^2^ (the most restricted), with higher values estimated for *K. laciniosa* and *K. rigida* (13,754 km^2^ and 19,256 km^2^, respectively). This disproportionately small core habitat indicates extreme ecological specificity, with optimal conditions realized only within narrow edaphic and microclimatic windows. Land cover analysis (Table S5) indicated that anthropogenic pressure was present in all suitability classes to varying degrees. Within the areas designated as core habitat, *K. scortechinii* had an exceptional 93.5% natural cover with only 6.5% anthropogenic influence, suggesting that its core habitat was largely within intact forest landscapes. In stark contrast, the core habitat of *K. flagellaris* and *K. rigida* exhibited significant anthropogenic pressure (51.6% and 50.6%, respectively), indicating that the most suitable areas of this species have been already heavily degraded and could need conservation. These estimates of anthropogenic pressure within core habitat, from *K. scortechinii* (largely intact) to *K. flagellaris* and *K. rigida* (severely degraded), reflects the species’ previously observed vulnerability. The high sensitivity of *K. scortechinii* to VPD and soil texture is consistent with its core habitat retaining high natural cover (93.5%), as these specific edaphic–climatic conditions coincide with remaining forest fragments. Conversely, *K. flagellaris’s* broader edaphic tolerances and lower VPD sensitivity placed its core habitat squarely within landscapes facing anthropogenic pressures. Very small core habitat areas estimated particularly for *K. flagellaris* (5563 km^2^) and *K. scortechinii* (980 km^2^), coupled with contrasting anthropogenic pressures, demand species-specific conservation strategies: protecting remnant intact forests for *K. scortechinii* while pursuing active restoration and habitat connectivity for *K. flagellaris* in already fragmented landscapes.

### 2.3. Characterization of Significant Environmental and Soil Variables at Species Locations

**Figure 6:**
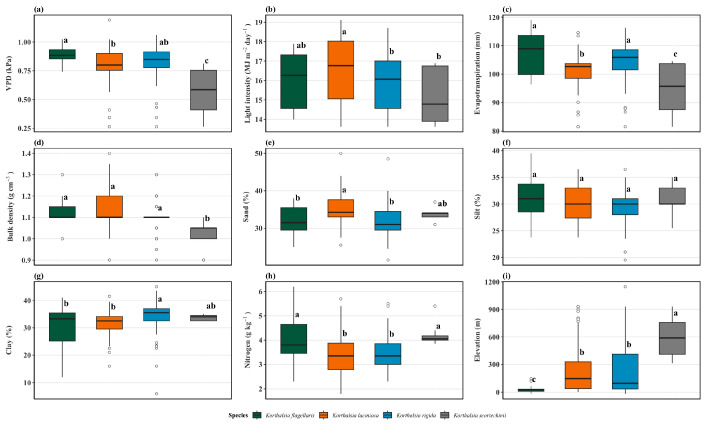
Boxplots comparing significant environmental and soil variables (as determined by the VIP scores of the ensemble SDM model) extracted at the four *Korthalsia* species locations. These include (**a**) vapor pressure deficit (VPD, kPa), (**b**) light intensity (MJ m^−2^ day^−1^), (**c**) evapotranspiration (mm), (**d**) bulk density (g cm^−3^), (**e**) sand percentage (%), (**f**) silt percentage (%), (**g**) clay percentage (%), (**h**) nitrogen content (g kg^−1^), and (**i**) elevation (m). Letters above the boxes indicate significant differences (*p* < 0.05). Unfilled circles beyond the whiskers are statistical outliers.

The variations in environmental and soil variables, extracting the species’ locations, are presented in Figure 6. *K. flagellaris* occupied the most humid location, characterized by the highest evapotranspiration and VPD, suggesting exposure to both through high atmospheric moisture demand. Locations of *K. laciniosa* were characterized by well-lit microsites and intermediate VPD. This combination suggests a strategy of exploiting canopy gaps with good light penetration but moist soils reducing evaporative demand. *K. rigida* generally occupied locations with lower light intensity and high evapotranspiration, implying growth in shaded microclimates with less atmospheric mixing. As observed previously, *K. scortechinii* exhibited the most distinct atmospheric conditions, with the lowest VPD and evapotranspiration, coupled with low light intensity, suggesting occurrence in environments with persistently low atmospheric demand, likely the most continuously humid, closed-canopy forests.

Consistent with climatic variation, soil properties also differed significantly among species, with locations of *K. scortechinii* having significantly less compact soils, exhibiting the lowest bulk density, a critical requirement for root penetration and aeration in humid understories. Soil texture patterns indicated that *K. laciniosa* was linked to sandier soils, consistent with its presence in well-drained, gap-phase microsites; *K. rigida* associated with locations having higher clay content, reflecting its preference for moisture-retentive soils. Notably, silt percentage did not differ among the four species, indicating that interspecific partitioning was driven primarily by sand-clay extremes. Soil nitrogen was significantly higher at locations of *K. flagellaris* and *K. scortechinii*, suggesting that these two species shared a requirement for greater nutrient availability despite occupying opposite ends of the VPD gradient. Lastly, *K. flagellaris* occurred at the lowest elevations (typically below 50 m) and *K. laciniosa* and *K. rigida* overlapped at mid-elevations, while *K. scortechinii* associated with the highest elevations among the four species. This elevational gradient further reinforced the species-specific atmospheric and edaphic windows, with lowland conditions favoring the high-VPD, nutrient-demanding *K. flagellaris* and upland settings providing the continuously humid, low-VPD conditions required by *K. scortechinii*.

## 3. Discussion

In this study, we modeled the potential distributions of four understudied *Korthalsia* species from the rattan genus in Southeast Asia to characterize their distribution and biogeographic patterns. While rattans are ecologically and economically vital to tropical forests, previous SDM studies have focused predominantly on a few commercially prominent genera such as *Calamus* in more seasonal environments using primarily climatic predictors [32,33]. This has left a significant gap in the understanding of the *Korthalsia* genus, a widespread yet poorly documented subgenus of rattan, and quantifying the environmental and soil influences on their distributions is a critical first step towards their evidence-based conservation planning in this rapidly changing region. The current SDM results indicated that distributions of these obligate climbing palms are collectively influenced by soil physical properties, atmospheric moisture demand, and canopy light availability.

### 3.1. SDM Modeling

The number of occurrences was highly variable, with *K. scortechinii* (*n* = 12) and *K. flagellaris* (*n* = 26) having the lowest number of observations. The unequal sample sizes (relative to *K. rigida* and *K. laciniosa*) either reflect a collection bias in Southeast Asian herbarium and GBIF records toward accessible lowland forest fragments [34] or a species rarity, thus imposing genuine limits on model confidence for these two species in under-sampled regions [35]. Among the models tested, GLM, GAM, GLMnet, and RF were the best performing classifiers (see Table 1), with RF having the highest classification metrics (highest AUC, COR, and TSS), highlighting its ability to handle complex environmental data and nonlinear interactions [36]. A post hoc spatial block cross-validation returned AUC values above 0.70 across species under a geographically independent evaluation, confirming that high training phase AUC values for the RF models were not inflated by spatial autocorrelation between training and test partitions [37].

The collinearity in the predictor set was determined based on a VIF threshold of <5, consistent with standard practice in SDM studies [38], to decrease model bias and improve classification accuracy [39]. Within the reduced set, the influence of atmospheric moisture demand, canopy light availability, and soil physical properties indicated a habitat requirement needed by the climbing palms to establish, elongate, and reproduce, rather than random dispersal patterns [36]. The physiological implications are thus constrained by soil texture, which governs root zone moisture retention; VPD, which governs the atmospheric cost of photosynthesis via stomatal regulation; and light availability, which governs the growth under gap-phase establishment, a result that is directly inferable from the VIP rankings prior to the mechanistic modeling.

The ensemble framework utilizing GLM, GAM, GLMnet, and RF with a VIF-based predictor selection (threshold < 5) consistently outperformed null models, proving particularly useful for data-limited species such as *K. scortechinii* and *K. flagellaris*. Relative to null models, full-model AUC values confirmed that the ensemble predictions captured genuine ecological signal in *Korthalsia* distributions. Ensemble estimates leverage the strengths of individual algorithms, reducing error, bias, and variance in classification [40]. The conservative predictions generated by the ensemble models, especially for data-limited taxa, more honestly reflect uncertainty in the underlying occurrence data, which is particularly valuable when modeling rare or poorly sampled species [35].

### 3.2. Species-Specific Modeling

The modeling indicated that despite sharing a broadly similar tropical Southeast Asian distribution, the four *Korthalsia* species exhibited pronounced partitioning along elevational, edaphic, and atmospheric moisture gradients including canopy light availability (light intensity), atmospheric moisture demand (VPD, evapotranspiration), and soil physical properties (sand, silt, clay). Climbing palms, including *Korthalsia*, can therefore be sensitive to canopy structure [41], hydrological conditions [42], as well as soil physical properties [43]. The composite suitability map (Figure 2) identified the Peninsular Malaysia, Borneo, and Sumatra as an area of high habitat suitability for the genus, consistent with known centers of rattan diversity [31].

The current observations of light being a key predictor in the presence of all species (as indicated by the SDM) aligns with the growth strategy of these climbing palms. Studies on rattans have highlighted their remarkable plasticity in response to environmental gradients, such as light availability [44]. This is specifically the case for climbing palms that cannot self-support and for which light availability directly constrains the establishment and vertical growth into the canopy gaps [45]. Climbing palms depend on host trees for structural support and cannot invest in self-supporting woody tissue [46] and must instead allocate carbon gains to rapid stem elongation toward canopy gaps [45]. Light availability in the understory therefore functions as a hard energetic limit on recruitment: where it is insufficient to support the carbon costs of elongation, establishment fails regardless of water or nutrient conditions. Additionally, light availability in the forest understory directly limits the photosynthetic carbon gain to support the stem elongation along the host trees, creating a direct physiological link between canopy gap dynamics and rattan distribution [44,47].

*K. laciniosa* exhibited a strong dependence on light availability, which may reflect a strategy of occupying forests with frequent canopy gaps [45,48]. In contrast, the presence of *K. rigida* and *K. flagellaris* was influenced by light conditions, with a previous study indicating dependence on canopy gaps in wet forests where gap-phase regeneration can be critical life history bottleneck [49]. The interspecific differences in light–moisture combinations imply that the effects of canopy disturbance, either through natural gap dynamics or anthropogenic fragmentation, would be species-specific, contingent on both the minimum light threshold and the moisture regime in which that light becomes accessible. *K. laciniosa* would be least negatively affected by disturbance that opens the canopy in drier sites, while *K. rigida* and *K. flagellaris* require canopy gaps specifically within humid forests.

The presence of *K. scortechinii* in low-VPD environments is consistent with understory palms sensitive to stomatal closure under atmospheric drought. In the current analysis, *K. scortechinii*, with the lowest mean VPD at locations where it is present (0.56 kPa), represents an extreme case of this hydraulic sensitivity among the four congeners. In contrast, the broader distribution of *K. laciniosa* reflects heliophilic plasticity (tolerance of a wide range of light intensities), tolerating the elevated evaporative demand in secondary and disturbed forests. Conversely, *K. flagellaris* was present in areas experiencing higher VPD, concurrently with high evapotranspiration, implying that stress resulting from atmospheric demand is physiologically manageable under sufficient soil moisture [50]. VPD as a dominant predictor for the distribution of *K. laciniosa* and *K. scortechinii* reflects the vulnerability of large-leaved climbing palms to atmospheric moisture deficit. Obligate climbing habit necessitates specialized physiological mechanisms to optimize photosynthesis, nutrient uptake, and water use efficiency, particularly under fluctuating environmental conditions [47]. Elevated VPD increases the leaf-to-air vapor pressure gradient, driving stomatal closure to prevent desiccation [51]. For slender-stemmed climbing palms with limited hydraulic capacitance, stomatal closure directly suppresses carbon assimilation and growth, even when soil moisture is adequate [52,53]. The low mean VPD at *K. scortechinii* locations is consistent with this mechanism, though the observed relationship is correlative rather than causative. Hence, VPD imposes a direct physiological cost on productivity in hydraulically vulnerable species.

The distribution of *K. flagellaris* was also found to be influenced by nitrogen as the top-ranked predictor, followed by elevation, which is well known to shape the vegetation structure and species diversity, particularly in rattan palms [54]. The influence of nitrogen availability supports sustained leaf production and photosynthetic enzyme synthesis required for rapid stem extension, a requirement that effectively confines *K. flagellaris* to fertile lowland soils, as observed in the current study through its core habitat being placed squarely within lowland landscapes facing active anthropogenic pressure (Table S5). A combination of high evapotranspiration and nitrogen influence indicates that *K. flagellaris* is associated with high-fertility substrates among the four congeners, whereas the other three species exhibited a stronger association with low-VPD environments.

Recent anthropogenic pressures have led to an abundance of rattans at higher elevations to avoid human disturbance [55]. It has been noted that anthropogenic activity in peat and forested swamp ecosystems [56], which support non-timber species such as *K. flagellaris*, face increasing pressure due to habitat loss from forestry expansion and peatland degradation, which threaten its natural populations and long-term viability [15]. As such, this degradation at lower elevations may alter the ecological dynamics for species like *K. flagellaris*, making effective management and conservation planning increasingly important.

The soil texture variables were highly influential in the distribution of the species as per the high VIP scores. Fine-textured soils with high silt and clay content retain moisture through capillary forces, providing a sustained water supply that buffers against atmospheric moisture demand—directly complementing the VPD signal. Coarser-textured soils drain rapidly and hold less water per unit volume, making them less effective at sustaining transpiration under elevated VPD. The interspecific differences in soil texture preferences, with *K. rigida* in clay-rich soils, *K. laciniosa* in sandier substrates, and *K. scortechinii* in coarse-textured soils, therefore reflect species-specific balances between drainage, aeration, and moisture retention requirements rather than arbitrary edaphic associations [10,33]. Soil texture may function as a primary driver of local-scale exclusion, and such edaphic specialization can be used as an actionable basis for plantation site matching and restoration planning [43].

A broad presence of *K. laciniosa* demonstrated environmental tolerance across a range of forest types as previously reported, including secondary and disturbed habitats [57]. This adaptability could be attributed to physiological and morphological traits related to light stress observed in the rattan genus [58]. *K. laciniosa* exhibited an affinity for sandier soil alignment, where rapid drainage prevents waterlogging during wet periods [59]. The importance of silt and clay percentage in the presence of *K. rigida* is suggestive of a preference for finer-textured soils with greater water-holding capacity, consistent with its occurrence in wet lowlands, characterized by moisture retention to buffer against short dry spells [60]. These edaphic partitioning patterns are consistent with observations of interspecific soil texture specialization in rattan communities across Southeast Asia, where fine-textured alluvial soils support distinct assemblages from coarser-textured upland substrates [10,33]. Soil texture may therefore function as a primary driver of local-scale exclusion, limiting congeneric competition among *Korthalsia* species at the microhabitat level.

In summary, this study provides the first ensemble SDM framework, establishing the first quantitative baseline distributional data for four *Korthalsia* species across Southeast Asia. By integrating atmospheric, hydrological, and edaphic predictors, we demonstrated that the distribution of these congeners is regulated by atmospheric moisture demand, canopy light availability, and soil texture, improving upon the previous studies about rattan distribution [32,33]. Furthermore, species-specific quantitative assessment of anthropogenic land cover within the predicted habitat classes provides spatially explicit information applicable for plantation site selection and restoration planning.

### 3.3. Limitations

Although the incorporation of species occurrence records with environmental variables represents a strong modeling framework, the limitations of available data must be acknowledged. Firstly, the modeling relied on publicly available occurrence data, which, despite rigorous cleaning, may incompletely represent the full environmental ranges of these species, particularly for *K. scortechinii*, which had the smallest number of locations. Secondly, the resolution of predictor layers, specifically the soil grids, may not fully capture the microsite variability relevant to understory species like rattans. Addressing these limitations through ground-truthing or field validation of predicted hotspots would increase the reliability of spatial modeling and improve its utility in conservation efforts [61]. Third, our models do not account for biotic interactions, including phorophyte availability. Although *Korthalsia* species are not documented as host-specific, their climbing habit requires structurally supportive trees of sufficient size and abundance [45]. Hence, future work that can integrating forest structure data would improve predictive accuracy. Given the sensitivity of these species to VPD, which is projected to intensify under warming scenarios [62], assessing climate vulnerability under future projected conditions represents an important next step that was beyond the scope of the current modeling framework. Finally, refining background sample selection using vegetation cover filters in future iterations would reduce commission errors in geographically proximate but ecologically unsuitable regions.

### 3.4. Implications for Conservation

Within the context of forest loss of wild rattan populations [63], the current findings provide empirical information for both the conservation and restoration of these species. The pronounced disparity between marginal and core habitat extents across all four species reflected the ecological specialization characteristic of obligate understory climbing palms, whose distribution within climatically suitable landscapes is likely further constrained by microhabitat requirements, including forest structure, canopy humidity, and host tree availability. Core habitat represented well under 1% of total suitable area across all four species (Table S4), acting as a *climatic refugia* (areas where localized microclimatic or edaphic conditions allow species to persist despite surrounding habitat modification) [64] that must be prioritized to prevent the functional extinction of these ecologically vital climbers. Under the extinction debt framework (which describes the future, inevitable extinction of species caused by past environmental changes (e.g., habitat loss, climate change)) [65], even modest additional land conversion within these areas translates nonlinearly into long-term population viability loss, a risk compounded by limited dispersal capacity in climbing palms.

A reduction in forested areas and overharvesting of wild rattan populations has been observed [19], with an urgent need for establishment and expansion of rattan plantations to supply raw material for the rattan-based industry [42]. Land cover analysis (Table S5) confirmed that anthropogenic modification was present within the core habitat for all four species, with the degree of modification varying substantially among species. Within the core habitat, *K. scortechinii* retained 93.5% natural cover with 6.5% anthropogenic pressure. In contrast, 51.6% and 50.6% of the core habitat was affected by anthropogenic pressure for *K. flagellaris* and *K. rigida*, respectively, indicating that the most suitable predicted habitat for these two species is already substantially modified. The relatively intact core habitat of *K. scortechinii* must be interpreted with caution given its extremely small extent (980 km^2^) and sparse occurrence data (n = 12) needing further ground validation, as stringent requirements with remaining forest fragments could be coincidental.

The soil texture preferences and light availability inferred in the current study can inform site selection and canopy management to optimize environmental suitability for conservation. Specifically, fine-textured moisture-retentive soils for *K. rigida* and *K. laciniosa*; nitrogen-rich, well-aerated lowland substrates for *K. flagellaris*; and persistently humid, low-VPD forest interior conditions with coarse-textured soils for *K. scortechinii* were derived from VIP rankings and species–location environmental characterization. Although only four out of approximately 26 known *Korthalsia* species were modeled, constrained by limited occurrence records, the four species examined represent a range of ecological patterns, from widespread (*K. flagellaris*, *K. laciniosa*) to narrow endemics with distinct traits (*K. rigida, K. scortechinii*), and several hold ethnobotanical value for cane and fiber [31], adding further relevance to understanding their environmental preferences. The findings of the current modeling framework can serve as an initial basis for identifying suitable restoration or plantation sites for these species.

## 4. Materials and Methods

### 4.1. Species Presence Data

**Figure 7:**
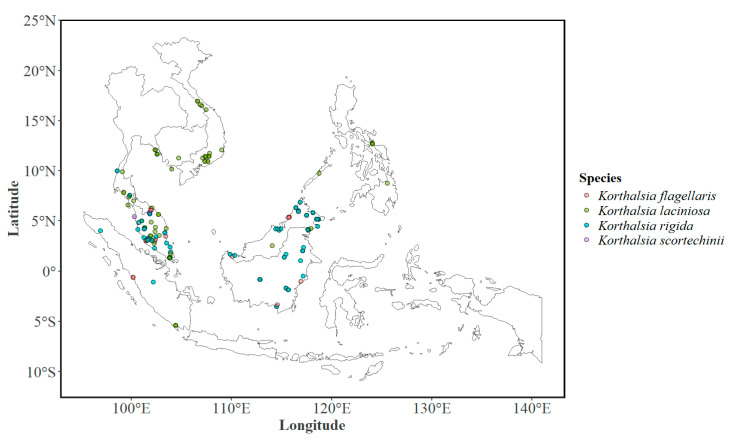
Distribution of the four *Korthalsia* species found in various countries of Southeast Asia (indicated by filled circles) as obtained from the public databases and personal observations [31] and supplemented by the locations reported by Shahimi et al. [10].

The geolocations of four *Korthalsia* species was determined using data from public databases that included the GBIF and the Botanical Information and Ecology Network (BIEN) [8,9,66]. The list was supplemented with the locations reported in the study by Shahimi et al. [10] and a few personal observations [31]. The datasets were also cross-referenced against the citizen science website iNaturalist at https://www.inaturalist.org/, and it was observed that the BIEN database did not contribute any additional presence records beyond those already available in the GBIF database. To avoid duplication, only the cleaned records obtained from the GBIF database were retained for modeling (the number of samples obtained through the data processing pipeline is presented in Table S6 and elaborated upon in Section 4.3). The rarity of species presence was evident through predominant occurrences being concentrated in Southeast Asia, indicating a preference for tropical and subtropical climates. Overall, 225 occurrences (see filled circles in Figure 7) around Southeast Asia were determined through the databases.

Species richness and phytogeographic affinities of the rattan genus *Korthalsia* in Malaysia, as documented in recent floristic assessments [10,31], provided an important supplementary dataset for refining species occurrence inputs. Data from this work were used to accentuate the number of known locations for *Korthalsia* species by incorporating additional verified records from underrepresented regions. These records, when integrated with GBIF and herbarium data, contributed to a more comprehensive spatial representation of species distributions across the Malaysian landscape, thereby improving the ecological and geographic breadth of the dataset used in model calibration.

### 4.2. Local Identification of Species

*Korthalsia* is a morphologically distinctive and well-defined genus, clearly separable from other rattans by a unique combination of vegetative and reproductive traits. Among *Korthalsia* species, vegetative characteristics, particularly leaflet shape, surface texture, and sheath features, are considered reliable, often allowing their identification even in the absence of reproductive material [67]. In this study, species identification was based on updated morphological descriptions and a new identification key developed specifically for species found in Thailand (*K. flagellaris*, *K. laciniosa*, *K. rigida*, and *K. scortechinii*) [31].

Each species exhibits a certain set of vegetative characters consistent across populations and distinguishable in field observations. For example, *K. flagellaris* and *K. laciniosa* can be differentiated through leaflet texture and the presence or absence of glaucous surfaces, while *K. rigida* is notable for its dense indumentum and distinct sheath features. *K. scortechinii* can be identified through its inflated ant-inhabited ocreas and myrmecophytic habit. To ensure accuracy and avoid false identifications, all specimens were cross-checked against herbarium vouchers and verified using both classic taxonomic treatments [67] and the revised key [31].

To define an ecologically sensible calibration region for species distribution modeling, the political boundaries were removed to obtain a merged area of all countries within tropical Southeast Asia, including Peninsular Malaysia, Borneo, Sumatra, Java, Thailand, Vietnam, Cambodia, and Laos. This ensured biogeographic continuity of the region given that *Korthalsia* species are not restricted by administrative borders. Dissolving national boundaries allowed background points to be drawn from a shared environmental space representative of the species’ potential range rather than being constrained by jurisdictional limits. While the calibration region included the broader tropical Southeast Asian landmass, background points were sampled only within terrestrial zones to avoid inclusion of oceanic areas. At this stage, no a priori filtering based on vegetation type or elevation was applied as the aim was to allow the models to define species–environment relationships from the occurrence data.

### 4.3. Occurrence Data and Sampling Design

To ensure the transparency and reproducibility of the modeling framework, the occurrence data assembly and filtering workflow were standardized across all four species. Primary occurrence records were synthesized from three major repositories: the Global Biodiversity Information Facility (GBIF), the citizen science platform iNaturalist, and peer-reviewed literature [10], supplemented by independent field observations [31]. The initial iNaturalist query, restricted to research-quality observations (georeferenced) with a coordinate uncertainty <10,000 m, yielded limited results (*K. flagellaris*: 1; *K. laciniosa*: 6; *K. rigida*: 4; *K. scortechinii*: 0). The initial GBIF query resulted in a total of 475 occurrences for the four species, as listed in Table S5. Following the consolidation of these sources and the removal of fossil specimens and records prior to the year 1800, as well as records with non-duplicated location information, the preliminary dataset comprised 102 occurrences across the four taxa (Table S6).

The data cleaning was done through a four-stage protocol to refine and supplement this initial dataset. First, exact duplicate records and those lacking precise geographic coordinates were excluded. Second, a spatial validation step was performed via intersection to ensure all records fell strictly within the defined Southeast Asian study extent. Third, to mitigate the risk of artificial inflation of model performance due to pixel-level clustering, a resolution-consistent filter was applied within the sdm framework. This step adjusted the presence data to the climate raster resolution, retaining only a single occurrence record per 1 km^2^ grid cell. After this resolution-based thinning, the cleaned dataset was reduced to 96 occurrences. This list was supplemented with relevant observations reported in Shahimi et al. [10] (88 occurrences) and a few personal observations [31] (41 occurrences), resulting in a final list of 225 total occurrences across the four species.

No additional distance-based spatial thinning was implemented beyond the resolution filtering. For restricted-range taxa such as *K. flagellaris* (n = 26) and *K. scortechinii* (n = 12), further reduction in sample size would have fallen below the threshold required for stable model convergence and reliable evaluation, a recognized trade-off when modeling rare taxa [35]. The resulting datasets represented the most comprehensive, spatially validated, and non-redundant records available, balancing the need for spatial independence with the statistical requirements of the ensemble modeling procedure.

### 4.4. Model Calibration and Pseudo-Absence Generation

To facilitate ensemble modeling, pseudo-absence points were generated to characterize the background environmental conditions of the study area. These points were generated using a spatially constrained random sampling approach, produced randomly across the Southeast Asian study extent using the sdmData() function within the sdm package [68], with the constraint that sampled locations were restricted to raster cells containing no occurrence records. In accordance with optimal sampling ratios for presence-background datasets, ten sets of pseudo-absences were generated at a 10:1 ratio relative to the number of presence records for each species [69].

To ensure spatial independence and reduce the risk of false absences, a spatial constraint was applied: pseudo-absences were restricted to raster cells with no occurrence points and were further filtered to exclude any locations within a 1 km buffer zone surrounding confirmed presence points. This buffering strategy minimizes spatial autocorrelation between presence and background data, thereby enhancing the discriminative power of the resulting models. No additional environmental stratification or bias correction was applied, as the random selection of background points across the entire study area ensures a representative sample of the available climate space, providing a robust baseline for evaluating habitat suitability [70].

### 4.5. Soil and Climate Data

The climate data rasters were downloaded from the TerraClimate global climate dataset [71] located at https://climate.northwestknowledge.net/TERRACLIMATE (accessed on 3 February 2025). The dataset has global coverage with a spatial resolution of 0.04^0^ degrees and a monthly temporal resolution, with monthly coverage during a period from 1958 to 2020. The spatial rasters included air temperature extremes (minimum or Tmin and maximum or Tmax), rainfall, downward shortwave surface radiation (light), vapor pressure deficit (VPD), wind speed, soil moisture content (in terms of volumetric content or VWC), evapotranspiration, and drought index (Palmer Drought Severity Index). The mean rasters of these parameters were calculated over a 10-year period from 2011 to 2020, corresponding to the most recent decade of complete data coverage available at the time of this analysis. All the data were accessed from the public domain and extracted and analyzed in the R statistical software [72].

The soil rasters were obtained from the SoilGrids dataset provided by the International Soil Reference and Information Centre or ISRIC [73] (located at https://files.isric.org/soilgrids/latest/data/). The dataset was downloaded and processed using the geodata and soil.world functions in R software (version 4.1.3). The database includes soil parameters at different soil depths such as soil organic carbon content, soil pH, sand, silt, and clay contents, bulk density, and cation-exchange capacity. Raster grids for selected soil properties that included bulk density; soil pH; sand, silt, and clay contents; and nitrogen content were downloaded at rooting depths observed for *Korthalsia* typical in forested environments between intervals of 0–5 cm and 5–15 cms and were averaged. Additionally, elevation raster (obtained using the elevatr package in R [74]) was also added to the SDM model. These rasters were further harmonized to have the same resolution as that of the climate variables using the projectRaster function in R through bilinear interpolation.

### 4.6. Species Distribution Model

Species distribution models (SDMs) explore relationships between species occurrences and variables that can influence their presence. Usually, these parameters include environmental and soil variables, with studies also using bioclim variables to analyze species distribution [75]. Such models are frequently used in determining richness of biodiversity, evolutionary biology, effect of invasive species, epidemiology, biology of global climate change, conservation, and wildlife management [76]. Multiple SDM fitting routines are available as integrated frameworks, which include openModeller [77], BIOENSEMBLES [78], and ModeEco [78], although the availability and maintenance status of such tools can change over time. In the current analysis, we use the sdm package developed by Naimi and Araújo [68] as it has a unified, reproducible workflow for fitting multiple SDM algorithms, generating ensemble predictions, and reporting performance using standardized evaluation outputs. The package employs an object-oriented structure that supports systematic model management and robust handling of failed runs across replicated partitions, and it also provides an optional graphical user interface to facilitate interactive setup and exploration of results.

The package includes several methods such as the Generalized Additive Model (GAM) [79], Generalized Linear Model (GLM) [80], Random Forest (RF) [81], and Boosted Regression Trees (BRTs) [82], Maximum Entropy (MaxEnt) [83] algorithms among others. All models were implemented using the default settings of the sdm package to ensure comparability in performance metrics. The sdm package internally manages certain aspects of model tuning. For instance, Random Forest uses an internal selection of optimal tree number and variable sampling, while MaxEnt applies its own internal feature selection and regularization procedures. No manual hyperparameter tuning was performed as the primary aim was to apply a consistent modeling protocol suitable for rare and data-limited species. Model significance was determined using null model testing [84], with full-model AUC values tested against the 95th percentile of the resulting null AUC distribution at *p* < 0.05.

### 4.7. Model Evaluation and Overfitting Mitigation

The climate and soil data rasters were used to generate a map of the most probable locations of *Korthalsia* species in Southeast Asia. To address the inherent risk of overfitting associated with complex machine learning algorithms like RF, model performance was validated using a multi-step protocol. First, excessive model complexity was mitigated by removing collinear environmental predictors with a Variance Inflation Factor (VIF) > 5 [85]. Second, model accuracy was determined using a subsampling approach where 30% of the occurrence data was withheld for independent testing in each of 10 replication runs. SDM performance accuracy was quantified using several performance metrics that included area under the curve (AUC), correlation coefficient (COR), true skill statistic (TSS), and deviance to determine the ability of the models to generalize to unseen data, with replication being implemented using both subsampling and bootstrap methods. These metrics were calculated for each iteration and then averaged to provide a summary of model performance. This multi-metric evaluation allowed for a comprehensive assessment of predictive ability, model fit, and reliability across different algorithms and species. For each species, models were replicated three times using random sampling under both methods (subsampling and bootstrap), with 30% of the data being randomly withheld for testing during each iteration.

As RF is prone to overfitting, to check if its performance was inflated due to spatial autocorrelation between training and test partitions, a post hoc spatial block cross-validation was carried out using the blockCV package [86]. Spatially independent folds (k = 5) were constructed using a block size of 200 km, ensuring geographic separation between training and test data in each fold [37]. The RF algorithm was refitted within each spatially independent training fold, and AUC was calculated on the geographically separated test fold. The mean test AUC across the five spatial folds was compared with the test sdm random cross-validation AUC to quantify the performance drop attributable to spatial autocorrelation.

Finally, to ensure ecological interpretations remained robust and were not driven by the specific fitting properties of a single algorithm, an ensemble modeling strategy was employed. An ensemble prediction [76,87], which combines the strengths of the selected algorithms, was then used to determine the species’ habitat suitability and distribution patterns [38]. Ensemble predictions were generated using the ensemble function provided within the sdm package. The weighted average of the contributions from the best performing models were calculated, effectively marginalizing potential overfitting in any single method and prioritizing consensus-based spatial predictions [35]. The consensus model was constructed by integrating predictions from multiple algorithms through a weighted ensemble of individual model predictions based on weights derived from their respective TSS scores. This modeling approach provided a balanced framework for ensemble prediction based on the relative reliability of each algorithm in classifying species’ presences from pseudo-absences as well as a comparative evaluation across the four *Korthalsia* species. This approach produced an ecologically meaningful distribution estimate across the four species.

### 4.8. Estimation of Suitability Areas and Anthropogenic Pressure

Potential suitable areas were calculated in km^2^ for each species by reclassifying the TSS-weighted ensemble suitability raster into three classes: Unsuitable (below the species-specific 10th percentile training presence threshold), Marginal Habitat (10th percentile to 0.40), and Core Habitat (>0.40), to provide spatially explicit estimates applicable to conservation planning and protected area assessment. Pixel areas were calculated using latitude-corrected cell sizes to account for the geographic coordinate system. Anthropogenic pressure within predicted suitable habitat was subsequently assessed to quantify the degree to which human land use has penetrated ecologically meaningful habitat zones—a critical consideration for evaluating the conservation status of data-poor taxa for which distributional information is otherwise limited. Land cover data were obtained from the GLC_FCS30D product [88] at 30 m global resolution for the year 2022, corresponding to the period of field observations. Land cover classes were aggregated into two ecologically meaningful categories: anthropogenic cover, comprising croplands, bare areas, and impervious surfaces; and natural cover, comprising tree cover and various forest types. The proportional area of each category within each suitability class was calculated per species and used to quantify the extent of human modification across the predicted habitat of each *Korthalsia* species. The land cover raster was applied as a post-modeling mask on the ensemble model to quantify the anthropogenic pressure within predicted habitat.

## 5. Conclusions

This study presents the first ensemble SDM framework for four *Korthalsia* species prevalent in Southeast Asia. VPD, light intensity, and soil texture variables (silt, clay, sand) were identified as the primary predictors of habitat suitability, reflecting a three-way influence of atmospheric moisture demand, canopy light availability, and edaphic physical properties that characterize the spatial distributions of these congeners. The modeled distributions indicated that *K. rigida* and *K. laciniosa* had broader environmental tolerances, while *K. scortechinii* and *K. flagellaris* had relatively specialized ecological requirement related to atmospheric moisture sensitivity and soil nitrogen dependency, respectively, as inferred from VIP scores and species–location environmental characterization. The core habitat represented less than 0.6% of total suitable area across all four species (Table S3), ranging from 980 km^2^ (*K. scortechinii*) to 19,256 km^2^ (*K. rigida*). Anthropogenic modification within the core habitat exceeded 50% for *K. flagellaris* (51.6%) and *K. rigida* (50.6%), while *K. scortechinii* retained 93.5% natural cover within a very limited extent of core habitat. These quantitative findings provide a species-specific spatial baseline for conservation prioritization, restoration planning, and plantation site selection based on the variables deemed significant in this study. Parts of Peninsular Malaysia and Sumatra represent priority areas for field validation of the predicted core habitat, particularly for *K. scortechinii* and *K. flagellaris*. Future work should assess climate vulnerability under projected VPD and temperature scenarios, as VPD was identified as the primary correlate of habitat suitability in the current analysis, and extend the framework to other undocumented *Korthalsia* species.

## Figures and Tables

**Table 1:** Mean test performance metrics of the top four species distribution modeling (SDM) routines, including AUC (area under the curve), COR (correlation), TSS (true skill statistic), and deviance, quantified through cross-validation (10 replicates, 30% test partition).

	Method	AUC	COR	TSS	Deviance
*K. rigida*	GLM	0.79	0.38	0.49	0.52
	GAM	0.88	0.57	0.62	0.54
	GLMnet	0.76	0.26	0.48	1.30
	RF	0.93	0.75	0.75	0.30
*K. flagellaris*	GLM	0.92	0.49	0.78	0.15
	GAM	0.84	0.52	0.68	1.29
	GLMnet	0.89	0.23	0.71	0.32
	RF	0.92	0.68	0.79	0.12
*K. laciniosa*	GLM	0.81	0.26	0.55	0.45
	GAM	0.87	0.51	0.69	1.19
	GLMnet	0.79	0.23	0.54	0.99
	RF	0.92	0.70	0.74	0.27
*K. scortechinii*	GLM	0.89	0.34	0.78	0.11
	GAM	0.88	0.60	0.76	0.62
	GLMnet	0.87	0.17	0.75	0.18
	RF	0.96	0.70	0.96	0.05

## Data Availability

Data used in the analyses can be found in the Supplementary Material.

## Supplementary Materials

The following supporting information can be downloaded at: https://www.mdpi.com/article/10.3390/plants15091348/s1. Table S1. Mean training performance metrics of the top four species distribution modeling (SDM) routines, including AUC (area under the curve), COR (correlation), TSS (true skill statistic), and deviance, quantified through cross-validation (10 replicates, 30% test partition). Note: GLM = Generalized Linear Models, GAM = Generalized Additive Models, GLMnet = Regularized Generalized Linear Models (Elastic Net), RF = Random Forest. Table S2. Comparison of sdm AUC and spatial block cross-validated AUC for the Random Forest algorithm across all four *Korthalsia* species. Spatial CV was conducted using blockCV (k = 5, block size = 200 km). Table S3. Null model significance test results for each *Korthalsia* species. All full model AUC values exceeded the 95th percentile of their respective null distributions (*p* < 0.05), confirming statistically significant model performance above random expectation. Figure S1. Null model AUC distributions. Histograms represent null model distribution over the various methods deemed as best performing per species. Black dashed line = mean null model AUC across all methods; red dashed line = mean full model AUC across all methods. The consistent separation between full model AUC and the null distribution confirms statistically significant predictive performance (*p* < 0.05) for all four species. Table S4. Potential suitable areas (km^2^) by suitability class for each *Korthalsia* species. Suitability classes were defined using three categories based on ensemble-predicted occurrence probability: Unsuitable (below the species-specific 10th percentile training presence threshold), Marginal Habitat (10th percentile threshold to 0.40), and Core Habitat (>0.40). The 10th percentile threshold was applied independently per species as a conservative criterion for delineating unsuitable from suitable habitat. Table S5. Land cover composition (%) within each suitability class. Land cover classes were aggregated into three categories: natural vegetation (broadleaved evergreen and deciduous tree cover, mixed forest, regularly flooded tree cover, and tree cover mosaics), anthropogenic cover (cultivated and managed areas, cropland mosaics, bare areas, and artificial surfaces), and other land cover (all remaining classes including shrubland, herbaceous cover, and water bodies). Percentages represent the proportion of total area within each suitability class occupied by each land cover category. Table S6. Summary of occurrence data assembly and filtering for the four *Korthalsia* species. The table details the number of records retrieved from global database (GBIF before checking with iNaturalist at https://www.inaturalist.org/), literature (Shahimi et al., 2023), and field observations, alongside the systematic reduction of data points through the cleaning process. Final sample sizes (n) represent the spatially independent occurrences used for ensemble niche modeling after resolution-consistent filtering elaborated in Section 4.3 of the manuscript.
