# Supplementary material for: Interspecific Habitat Suitability of Four Southeast Asian Spiny Climbing Palms (Korthalsia) Through Species Distribution Modeling
Source: Plants (Basel). 2026 Apr 28;15(9):1348. doi: 10.3390/plants15091348 (PMC13164649; doi:10.3390/plants15091348)
Supplement: Supplementary file 1 [file plants-15-01348-s001.zip › plants-4188678-supplementary.pdf]

Species	Method	AUC	COR	TSS	Deviance
K. rigida	GLM	0.81	0.42	0.49	0.49
	GAM	0.93	0.71	0.73	0.30
	GLMnet	0.78	0.27	0.47	1.28
	RF	0.96	0.97	1.00	0.10
K. flagellaris	GLM	0.95	0.61	0.80	0.11
	GAM	0.99	0.97	0.98	0.01
	GLMnet	0.93	0.25	0.76	0.29
	RF	0.98	0.97	1.00	0.03
K. laciniosa	GLM	0.84	0.32	0.59	0.43
	GAM	0.97	0.78	0.87	0.17
	GLMnet	0.82	0.26	0.56	1.01
	RF	0.98	0.97	1.00	0.09
K. scortechinii	GLM	0.92	0.46	0.79	0.08
	GAM	1.00	1.00	1.00	0.00
	GLMnet	0.89	0.16	0.75	0.16
	RF	1.00	0.97	1.00	0.02

Table S1. Mean training performance metrics of the top four species distribution modeling (SDM) routines, including AUC (area under the curve), COR (correlation), TSS (true skill statistic), and deviance, quantified through cross-validation (10 replicates, 30% test partition).
Note: GLM = Generalized Linear Models, GAM = Generalized Additive Models, GLMnet = Regularized Generalized Linear Models (Elastic Net), RF = Random Forest.

**Species**	**Random AUC (Test)**	**Spatial CV RF AUC (Test)**	**SD (Spatial CV)**	**Drop In AUC performance**
K. rigida	0.93	0.73	0.095	0.20
K. flagellaris	0.92	0.71	0.036	0.21
K. laciniosa	0.92	0.74	0.082	0.18
K. scortechinii	0.98	0.81	0.094	0.17

Table S2. Comparison of sdm AUC and spatial block cross-validated AUC for the Random Forest algorithm across all four *Korthalsia* species. Spatial CV was conducted using blockCV (k = 5, block size = 200 km).

**Species**	**Null Model Mean_AUC**	**Full Model Mean_AUC**
K. rigida	0.504	0.842
K. flagellaris	0.507	0.892
K. laciniosa	0.493	0.848
K. scortechinii	0.534	0.905

Table S3. Null model significance test results for each *Korthalsia* species. All full model AUC values exceeded the 95th percentile of their respective null distributions ($p < 0.05$), confirming statistically significant model performance above random expectation.

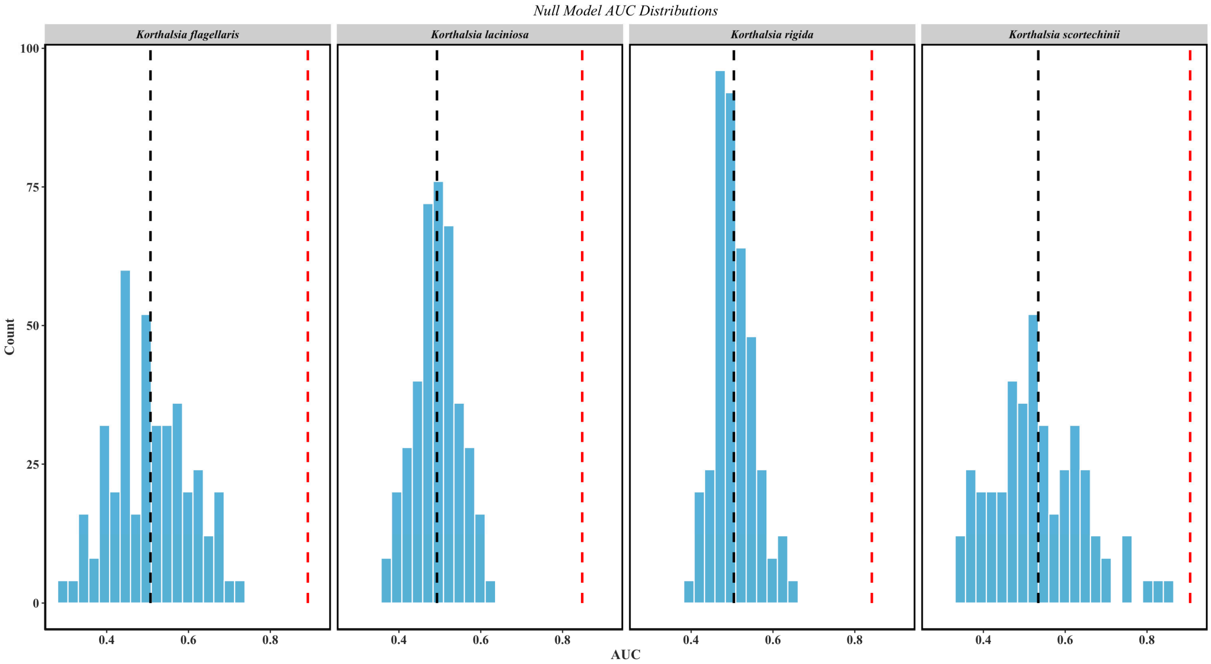

Figure S1. Null model AUC distributions. Histograms represent null model distribution over the various methods deemed as best performing per species. Black dashed line = mean null model AUC across all methods; red dashed line = mean full model AUC across all methods. The consistent separation between full model AUC and the null distribution confirms statistically significant predictive performance ($p < 0.05$) for all four species.

Species	Unsuitable	Marginal Habitat	Core Habitat
K. rigida	344,461	3,164,184	19,256
K. flagellaris	346,088	3,176,249	5,563
K. laciniosa	349,149	3,164,998	13,754
K. scortechinii	344,986	3,181,934	980

Table S4. Potential suitable areas (km^2) by suitability class for each *Korthalsia* species. Suitability classes were defined using three categories based on ensemble-predicted occurrence probability: Unsuitable (below the species-specific 10th percentile training presence threshold), Marginal Habitat (10th percentile threshold to 0.40), and Core Habitat (>0.40). The 10th percentile threshold was applied independently per species as a conservative criterion for delineating unsuitable from suitable habitat.

Species	Suitability Class	Natural (%)	Anthropogenic (%)	Other (%)
K. rigida	Unsuitable	18.6	62.0	19.3
	Marginal Habitat	47.6	38.7	13.7
	Core Habitat	31.6	50.7	17.8
K. flagellaris	Unsuitable	39.2	38.8	22.0
	Marginal Habitat	45.3	41.3	13.4
	Core Habitat	24.8	51.6	23.6
K. laciniosa	Unsuitable	27.0	57.6	15.4
	Marginal Habitat	46.7	39.3	14.1
	Core Habitat	29.1	35.0	35.9
K. scortechinii	Unsuitable	17.2	64.6	18.2
	Marginal Habitat	47.6	38.5	13.9
	Core Habitat	93.5	6.5	0.0

Table S5. Land cover composition (%) within each suitability class. Land cover classes were aggregated into three categories: natural vegetation (broadleaved evergreen and deciduous tree cover, mixed forest, regularly flooded tree cover, and tree cover mosaics), anthropogenic cover (cultivated and managed areas, cropland mosaics, bare areas, and artificial surfaces), and other land cover (all remaining classes including shrubland, herbaceous cover, and water bodies). Percentages represent the proportion of total area within each suitability class occupied by each land cover category.

Species	iNaturalist	Pre-processed GBIF	Raw GBIF (records with locations)	Post-Filtering GBIF (records without duplicated locations)	Personal observations	Shahimi et al. (2023)	Final n
K. rigida	4	192	41	40	13	56	109
K. laciniosa	6	208	48	45	14	19	78
K. flagellaris	1	52	9	8	5	13	26
K. scortechinii	0	23	4	3	9	0	12
**Total**	11	475	102	96	41	88	**225**

Table S6. Summary of occurrence data assembly and filtering for the four *Korthalsia* species. The table details the number of records retrieved from global database (GBIF before checking with iNaturalist at https://www.inaturalist.org/), literature (Shahimi et al., 2023), and field observations, alongside the systematic reduction of data points through the cleaning process. Final sample sizes (n) represent the spatially independent occurrences used for ensemble niche modeling after resolution-consistent filtering elaborated in section 4.3 of the manuscript.
